# Chylomicron-Derived Fatty Acid Spillover in Adipose Tissue: A Signature of Metabolic Health?

**DOI:** 10.1210/jc.2017-01517

**Published:** 2017-11-01

**Authors:** Marie-Eve Piché, Siôn A. Parry, Fredrik Karpe, Leanne Hodson

**Affiliations:** 1 Oxford Centre for Diabetes, Endocrinology and Metabolism, University of Oxford, Churchill Hospital, Oxford OX3 7LE, United Kingdom; 2 Quebec Heart and Lung Institute, Laval University, Quebec G1V 4G5, Canada; 3 National Institute for Health Research Oxford Biomedical Research Centre, Oxford University Hospital Trusts, Oxford OX3 9DU, United Kingdom

## Abstract

**Context and Objectives:**

Spillover of fatty acids (FAs) into the plasma nonesterified fatty acid (NEFA) pool, because of an inability of adipose tissue (AT) to accommodate sufficient fat uptake, has been suggested to contribute to obesity-related insulin resistance. Using specific labeling techniques, we compared the proportion of spillover-derived NEFA across a range of adiposity.

**Participants and Methods:**

Seventy-one healthy men and women were fed a mixed meal (40 g fat) containing [U^13^C]palmitate to assess the contribution of chylomicron-derived spillover FAs. To investigate subcutaneous abdominal-specific spillover, arteriovenous difference and stable-isotope methodologies were used in substudy (six men, six women).

**Results:**

Chylomicron-derived FA spillover was higher in individuals with a BMI <25 kg/m^2^ (n = 18) compared with those with a BMI ≥25 kg/m^2^ (n = 53) (22.2 ± 1.6% vs 18.6 ± 0.7%, *P* = 0.02). Women had higher chylomicron-derived FA spillover than age- and BMI-matched men (21.9 ± 1.1% vs 15.0 ± 1.6%, *P* = 0.001). Assessing spillover across subcutaneous abdominal AT showed higher proportions in women than in men (28.5 ± 6.1% vs 9.9 ± 1.3%, *P* = 0.01).

**Conclusion:**

There is a considerable degree of spillover FA into the systemic NEFA pool in the postprandial state; this process is greater and more dynamic in lean individuals and women. Contrary to general perception, spillover of chylomicron-derived FA into systemic circulation is a physiologically normal feature most easily observed in people with a higher capacity for clearance of plasma triglycerides, but does not appear to be a pathway providing excess NEFA in obesity.

## Teaser

The proportion of diet-derived spillover fatty acids was assessed in healthy adults. There was a more dynamic and greater degree of postprandial spillover of fatty acids in lean individuals and women.

Subcutaneous adipose tissue (AT) plays a central role in the regulation of fatty acid (FA) release and storage in the fasting and postprandial states with AT responding dynamically to alterations in nutritional state (1, 2). Expansion of AT depots, as seen in obesity, may contribute to altered FA trafficking. It has been proposed that poorly controlled release of nonesterified FA (NEFA) from AT underlies the metabolic derangements observed in obese and insulin-resistant individuals (3).

In the transition from the postabsorptive to postprandial state, chylomicrons containing triglycerides (TGs) from dietary fat require hydrolysis by lipoprotein lipase (LPL) (4, 5). It has been suggested LPL acts preferentially on TG in chylomicrons rather than TG in very low density lipoprotein (VLDL) (6). Postprandial studies have demonstrated that a proportion of FAs liberated by the action of LPL on chylomicron-TG, are not taken up by AT, rather they spillover into the systemic NEFA pool and may contribute to postprandial NEFA concentrations (3). Under extreme conditions of restricted AT FA storage, such as partial lipodystrophy (7), chylomicron-derived FAs appear to provide a substantial contribution to systemic NEFA concentrations.

Information regarding the effect of adiposity and sex on the proportion of spillover FAs entering systemic circulation is sparse. It has been speculated that chylomicron-derived FA spillover is due to an inability of the obese/insulin-resistant AT to accommodate sufficient fat uptake and thus contributes to augmentation of insulin resistance and channelling of FAs toward ectopic fat depots (3). Therefore, we have investigated the contribution of chylomicron-derived spillover FAs into the systemic NEFA pool in apparently healthy men and women across a range of adiposity, after consumption of a single, standardized mixed test meal, with the use of specific labeling techniques. We also explored, in a subgroup of participants, AT-specific chylomicron-derived FA spillover using arteriovenous difference and stable-isotope methodologies.

## Subjects and Methods

### Participants

Seventy-one subjects were recruited from the Oxford BioBank (8) or locally by advertisement. All subjects were free from known disease, nonsmoking, not taking medication known to affect glucose and lipid metabolism, and did not consume alcohol above recommended limits (9). The studies were approved by the Oxfordshire and Portsmouth Clinical Research Ethics Committees. All subjects gave written informed consent. Some of the data, but not all, reported in this work, constitute a reanalysis of previously published studies (2, 10–14).

### Study protocol

The study protocol has been previously described (2, 12). Briefly, all participants were asked to refrain from alcohol and strenuous exercise for 24 hours before the study day. Participants arrived in the clinical research unit after an overnight fast. After a baseline blood sample was taken, participants were fed a standard test meal (40 g fat, 40 g carbohydrate) containing a fixed (equivalent to 100 mg) dose of [U^13^C]palmitic acid to trace the fate of dietary FAs. After consumption of the meal, blood samples were taken at regular intervals over the following 360 minutes. Fat mass was calculated from bioelectric impedance (2, 10, 11, 13) or, in later studies, by dual-energy X-ray absorptiometry (12, 14).

### Analytical methods

Whole blood was collected into heparinized tubes (Sarstedt, Leicester, United Kingdom) and plasma immediately separated by centrifugation for the measurement of plasma insulin, glucose, NEFA, and TG (2, 15).

Separation of chylomicrons [Svedberg flotation rate (S_f_) >400] and VLDL-rich fractions (S_f_, 20 to 400) were made by sequential flotation using density gradient ultracentrifugation (16). The S_f_ 20 to 400 fraction was then further separated by immunoaffinity chromatography as described (17).

Samples were taken at baseline (0 minutes) and, 30, 60, 90, 120, 180, 240, 300, and 360 minutes after the mixed test meal for plasma biochemistry and stable-isotope enrichment and at 0, 120, 280, 240, 300, and 360 minutes for the analysis of chylomicron and VLDL fractions. At each blood sampling time point, a breath sample was collected into EXETAINER® tubes (Labco Ltd, High Wycombe, United Kingdom) for ^13^CO_2_ enrichment. Oxygen consumption and carbon dioxide production were measured by using a ventilated-hood indirect calorimeter (Deltatrac; Datex, Helsinki, Finland, or the Gas Exchange Measurement analyzer; GEMNutrition Ltd, Cheshire, United Kingdom) in the fasting and postprandial (120 minutes) states.

### Arteriovenous difference study

To determine the LPL-derived FA release from subcutaneous AT, we studied a subgroup of participants (n = 12, 6 men and 6 women). Subcutaneous abdominal AT arteriovenous difference measurements were made as described (11). Arterial or arterialized venous blood samples, along with venous effluent of subcutaneous abdominal AT, were taken simultaneously at baseline (0 minutes) and at 150 minutes after the meal for some [(10) and unpublished data] and at 120 and 180 minutes for other participants (2, 11, 13). For the latter, we averaged the data between these two time points.

### Fatty acid analysis and isotopic enrichment

To determine the specific FA composition and isotopic enrichment, total lipids were extracted from plasma and lipoproteins fractions and FA methyl esters prepared as described (17). FA compositions (µmol/100 µmol total fatty acids) were determined by gas chromatography (17) and palmitate concentrations were calculated (15).

### Analysis of [U^13^C]palmitate enrichments

[U^13^C]palmitate enrichments in plasma NEFA and TG fractions were determined by gas chromatography mass spectrometry (2). Tracer-to-tracee ratio (TTR) for [U^13^C]palmitate (M+16)/(M+0) was calculated and multiplied by the corresponding palmitate-NEFA or palmitate-TG concentration to give tracer concentrations. The TTR of a baseline measurement (before tracer administration) was subtracted from each sample TTR to account for natural abundance. Analysis of ^13^C enrichment in breath CO_2_ samples and the relative rate of whole-body meal-derived FA oxidation was calculated by multiplying the carbon dioxide production (mmol/min) by the TTR of ^13^CO_2_/^12^CO_2_, as described (18), and corrected for individual lean mass.

### Calculations and statistical analysis

Insulin resistance was assessed with the homeostasis assessment model of insulin resistance (HOMA-IR) (19).

The relative contribution of systemic chylomicron-derived spillover FAs to the plasma NEFA pool was calculated as: TTR of [^13^C]palmitate in the systemic NEFA pool divided by peak chylomicron-TG TTR (20).

Fractional extraction was calculated as the arteriovenous difference divided by arterial TG concentration and expressed as a percentage (2). The proportion of AT-derived spillover FA was calculated as the venous-arterial difference in the labeled NEFA divided by the arteriovenous difference in labeled TG (10).

Data were analyzed using SAS software, version 9.4 (SAS Institute Inc, Cary, NC). All data are presented as means ± standard error of the mean unless otherwise stated. Areas under the curve (AUCs) were calculated by the trapezoid method and divided by the relevant time period to give time-averaged values. Normality of data distribution was verified using normality tests (Shapiro-Wilk, Kolmogorov-Smirnov), skewness, and kurtosis statistics. Anthropometric and biochemical measures were compared between groups using a one-way analysis of variance followed by Student *t* (two-sided) or Mann-Whitney tests. Comparisons across different time periods between groups were analyzed by repeated measures two-way analysis of variance followed by the Holm-Sidak post hoc test. Statistical significance was set at *P* < 0.05. Associations between chylomicron-derived FA spillover with anthropometric and metabolic variables were carried out using Spearman rank correlation. Multiple stepwise linear regression was performed to explore which variable independently correlated with chylomicron-derived FA spillover. Sex, body mass index (BMI), HOMA-IR, and TG levels showing a *P* ≤ 0.05 in the correlation analysis were introduced in the stepwise selection process.

## Results

### Effect of adiposity on chylomicron-derived FA spillover

The BMI of participants ranged from 19.5 to 34.8 kg/m^2^. Participants were classified as having a low (<25 kg/m^2^) or high (≥25 kg/m^2^) BMI; there was no difference in the age or proportion of women between the groups (Table 1). As expected, individuals with a high BMI had a significantly higher waist circumference and total body fat content (all *P* < 0.001) compared with individuals with a low BMI (Table 1). HOMA-IR and fasting plasma insulin concentrations were significantly (*P* < 0.05) higher in the high compared with the low BMI group (Table 1).

**Table 1.** **Participant Characteristics According to BMI**

	**Low BMI (<25.0 kg/m^2^) (n = 18)**	**High BMI (≥25.0 kg/m^2^) (n = 53)**
Age (y)	41.4 ± 2.8	43.5 ± 0.9
Female sex (n)	6	10
BMI (kg/m^2^)	22.7 ± 0.4	28.5 ± 0.3^*a*^
Waist circumference (cm)	82.3 ± 1.7	97.3 ± 1.2*^a^*
Waist-to-hip ratio	0.86 ± 0.01	0.92 ± 0.02*^a^*
Body fat (%)	22.7 ± 2.4	31.1 ± 1.1*^a^*
HOMA-IR	2.24 ± 0.22	3.07 ± 0.17*^b^*
Fasting plasma biochemical parameters		
Glucose (mmol/L)	5.2 ± 0.1	5.3 ± 0.1
Insulin (mU/L)	9.6 ± 0.8	12.8 ± 0.7*^b^*
TG (mmol/L)	1.27 ± 0.15	1.72 ± 0.13
VLDL-TG (µmol/L)	416 ± 45	493 ± 40
NEFA (µmol/L)	498 ± 38	483 ± 24
Time-averaged postprandial plasma biochemical parameters		
Glucose (mmol/L)	5.5 ± 0.1	5.5 ± 0.1
Insulin (mU/L)	22.4 ± 2.4	28.5 ± 1.9
TG (mmol/L)	1.9 ± 0.2	2.4 ± 0.2
NEFA (µmol/L)	402 ± 26	382 ± 12

Data are presented as mean ± standard error of the mean.

^a^ *P* < 0.001 and *^b^P* < 0.05 low (<25 kg/m^2^) vs high BMI (≥25.0 kg/m^2^).

Although fasting plasma insulin concentrations were significantly different, there was no difference in the postprandial concentrations of plasma glucose, insulin, TG, or NEFA between individuals with a low or high BMI (Table 1).

**Figure 1.**
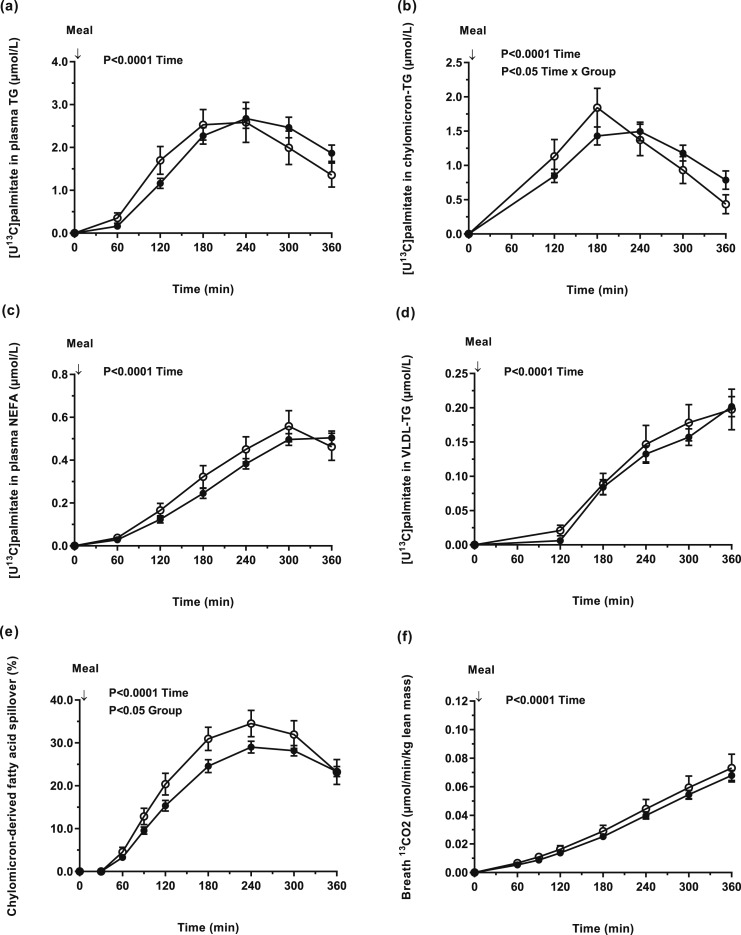
Concentrations of dietary [U^13^C]palmitate in (a) plasma TG (effect of time, *P* < 0.0001); (b) chylomicron-TG (effect of time, *P* < 0.0001; time × group interaction, *P* = 0.04); (c) NEFA (effect of time, *P* < 0.0001); (d) VLDL-TG (effect of time, *P* < 0.0001); (e) the contribution of chylomicron-derived FA spillover to the plasma NEFA pool (effect of time, *P* < 0.0001; group, *P* = 0.02); and (f) the appearance of [^13^C] in breath CO_2_ (per unit lean mass) (effect of time, *P* < 0.0001) in participants with low BMI (<25 kg/m^2^) (n = 18) (white circle) and high BMI (≥25 kg/m^2^) (n = 53) (black circle).

By feeding a mixed test meal containing [U^13^C]palmitate, we were able to trace the fate of dietary fat. We found no difference in the appearance of [U^13^C]palmitate in plasma TG, chylomicron-TG, plasma NEFA, or VLDL-TG between individuals with a low or high BMI [Figs. 1(a)–1(d)]. We then calculated the relative contribution of chylomicron-derived spillover FA to the systemic NEFA pool and found it to be significantly (*P* < 0.05) higher in individuals with a low compared with high BMI [Fig. 1(e)]. The AUC (time-averaged) for chylomicron-derived spillover FA was higher in the low compared with high BMI group (22.2 ± 1.6% vs 18.6 ± 0.7%, *P* < 0.05). We assessed whole-body dietary FA oxidation by measuring the incorporation of ^13^C (from dietary fat) in breath CO_2_ and found no difference between the groups [Fig. 1(f)]. Because chylomicron-derived FA spillover may be higher in obese individuals, we classified individuals as nonobese (BMI < 30 kg/m^2^, n = 56) or obese (BMI ≥ 30 kg/m^2^, n = 14). We found chylomicron-derived FA spillover was significantly lower in obese compared with nonobese individuals (16.9 ± 1.1% vs 19.9 ± 0.7%, *P* < 0.05), respectively. Because fat mass may play an important role in FA spillover, we compared the contribution of chylomicron-derived spillover FAs in the systemic NEFA pool according to fat mass and using the median fat mass % for females (39.1%) and males (27.3%) as cutoffs. We found no difference between women with a fat mass <39.1% vs ≥39.1% (23.3 ± 1.8 vs 21.8 ± 1.6%) and men with a fat mass <27.3% vs ≥27.3% (19.6 ± 1.2 vs 17.6 ± 0.8%), respectively.

### Effect of sex on chylomicron-derived FA spillover

To compare the effects of sex on chylomicron-derived FA spillover, we matched women and men by age and BMI (Table 2). As expected, women had a smaller waist circumference (*P* < 0.05) and waist-to-hip ratio, but greater total body fat content (*P* < 0.001) than men (Table 2). Despite the significant difference in body fat (38 ± 2% vs 26 ± 1%, women vs men, *P* < 0.0001), fasting plasma NEFA concentrations were not different; women had significantly (*P* < 0.01) lower fasting plasma glucose, TG, and VLDL-TG concentrations compared with men (Table 2). Differences in fasting plasma biochemical parameters between the groups were maintained over the postprandial period; there was no difference in postprandial plasma insulin or NEFA concentrations (Table 2).

**Table 2.** **Participant Characteristics and Fasting Plasma Metabolites Concentrations**

	**Women (n = 15)**	**Men (n = 15)**
Age (y)	43.5 ± 2.2	43.0 ± 2.4
BMI (kg/m^2^)	26.4 ± 0.9	26.8 ± 1.1
Waist circumference (cm)	86.7 ± 2.5	97.0 ± 3.5*^a^*
Waist-to-hip ratio	0.84 ± 0.01	0.94 ± 0.02*^b^*
Body fat (%)	38.4 ± 2.3	25.5 ± 2.8*^c^*
HOMA-IR	2.30 ± 0.36	3.42 ± 0.38*^a^*
Fasting plasma biochemical parameters		
Glucose (mmol/L)	5.0 ± 0.1	5.5 ± 0.1*^c^*
Insulin (mU/L)	10.2 ± 1.5	13.8 ± 1.4
TG (mmol/L)	0.9 ± 0.1	2.1 ± 0.3*^c^*
VLDL-TG (µmol/L)	305 ± 46	579 ± 92*^c^*
NEFA (µmol/L)	508 ± 37	492 ± 48
Time-averaged postprandial plasma biochemical parameters		
Glucose (mmol/L)	5.1 ± 0.1	5.7 ± 0.2*^c^*
Insulin (mU/L)	23.9 ± 3.5	32.1 ± 4.5
TG (mmol/L)	1.44 ± 0.21	2.67 ± 0.33*^c^*
NEFA (µmol/L)	372 ± 19	415 ± 27

Data are presented as mean ± standard error of the mean. Women and men were matched according to age (±2 years) and BMI (±3 kg/m^2^).

^a^ *P* < 0.05, *^b^P* < 0.001, *^c^P* < 0.01, women vs men.

**Figure 2.**
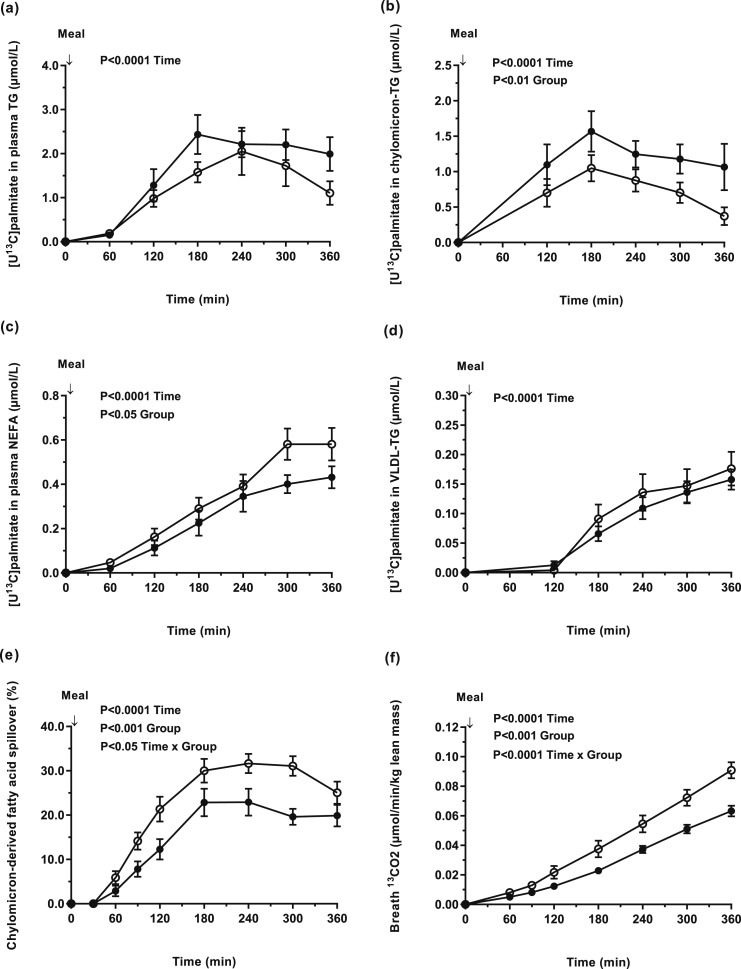
Concentrations of dietary [U^13^C]palmitate in (a) plasma TG (effect of time, *P* < 0.0001); (b) chylomicron-TG (effect of time, *P* < 0.0001; group, *P* = 0.01); (c) plasma NEFA (effect of time, *P* < 0.0001; group, *P* = 0.05); (d) VLDL-TG (effect of time, *P* < 0.0001); (e) the contribution of chylomicron-derived fatty acid spillover to the plasma NEFA pool (effect of time, *P* < 0.0001; group, *P* = 0.0009; time × group interaction, *P* = 0.02); and (f) the appearance of [^13^C] in breath CO_2_ (per unit lean mass) (effect of time, *P* < 0.0001; group, *P* = 0.0004 and time × group interaction, *P* < 0.0001) in BMI-matched women (n = 15) (white circle) and men (n = 15) (black circle).

The concentration of [U^13^C]palmitate in plasma chylomicron-TG was significantly higher in men compared with women (*P* < 0.05) [Fig. 2(b)], whereas there was no difference in [U^13^C]palmitate concentrations in plasma TG and VLDL-TG between groups [Fig. 2(a) and 2(d)]. In contrast, the concentration of [U^13^C]palmitate in the systemic NEFA pool was significantly (*P* < 0.05) higher in women compared with men [Fig. 2(c)]. The relative contribution of chylomicron-derived spillover FA to the systemic NEFA pool was higher (*P* < 0.05) for women compared with men; chylomicron-derived spillover FA accounted for 30% of the plasma NEFA pool in women at 240 minutes following consumption of the mixed test meal [Fig. 2(e)]. The appearance of [^13^C] in breath CO_2_ (per unit lean mass) was greater in women compared with men (*P* < 0.01) [Fig. 2(f)].

The sex differences in fasting plasma TG and HOMA-IR (Table 2) are in line with our recent observations (8). When matched for these variables, chylomicron-derived FA spillover remained higher (*P* = 0.05) in women (n = 14) compared with men (n = 14) (data not shown).

### Subcutaneous abdominal AT chylomicron-derived FA spillover

**Figure 3.**
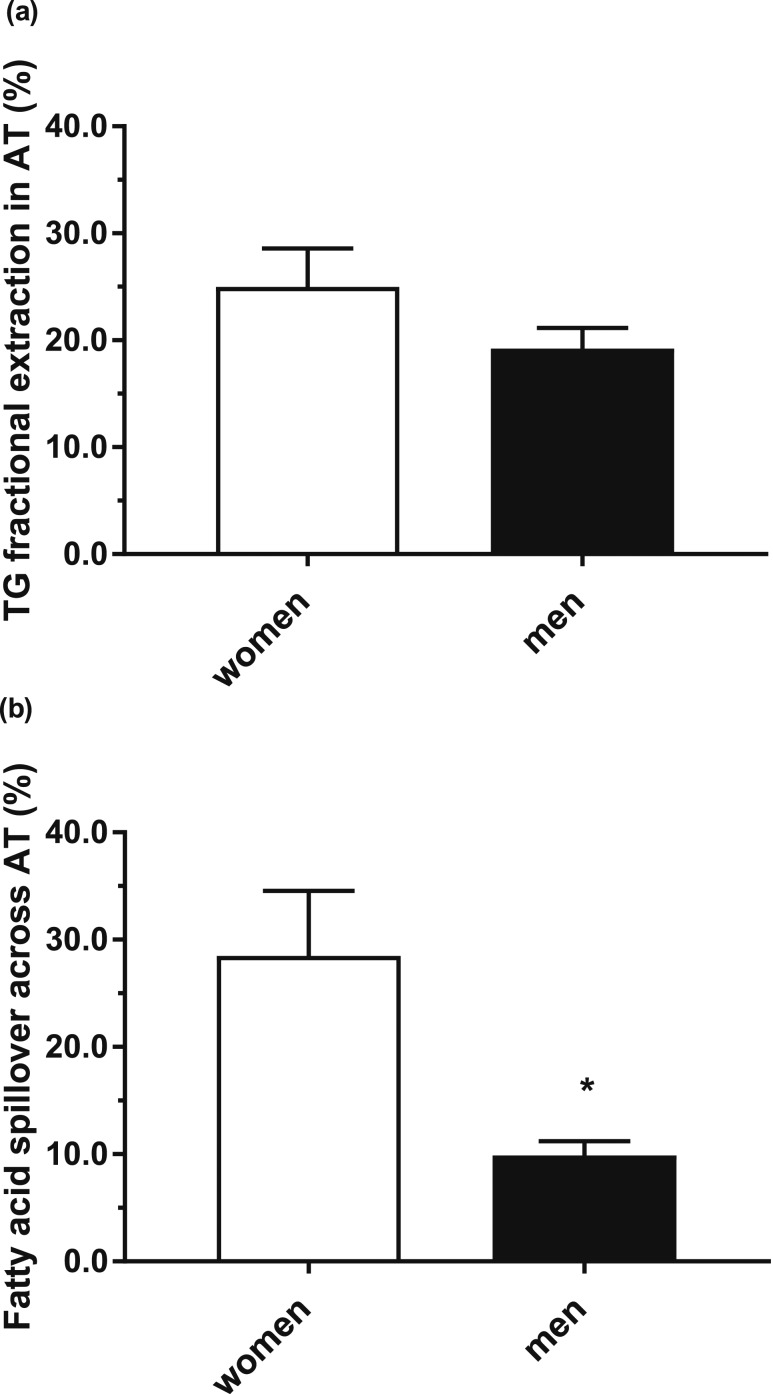
The movement of fatty acids across subcutaneous abdominal AT (a) postprandial TG fractional extraction across subcutaneous AT and (b) chylomicron-derived fatty acid spillover across subcutaneous abdominal AT in women (n = 6) (white bar) and men (n = 6) (black bar). *P < 0.01 women vs men.

To precisely define the FA movement across subcutaneous abdominal AT, we conducted a subgroup analysis of participants (6 women and 6 men) who had undergone arteriovenous blood sampling. The mean age and BMI of women and men did not differ (38.3 ± 4.2 years vs 37.8 ± 4.3 years and 22.9 ± 0.4 kg/m^2^ vs 23.4 ± 0.6 kg/m^2^, women vs men, respectively). Women had a significantly (*P* < 0.05) lower waist circumference but significantly (*P* < 0.01) more body fat than men (28.3 ± 2.0% vs 17.6 ± 1.6%). There were no differences in fasting plasma glucose, insulin, NEFA, or TG concentrations between the sexes (data not shown). We calculated the fractional extraction (%) of dietary fat across AT at 150 minutes after consumption of the mixed test meal and found that, although higher in women, it was not significantly different from men (25.0 ± 3.6% vs 19.2 ± 1.9%, women vs men, *P* = 0.19) [Fig. 3(a)]. We then determined the proportion of chylomicron-derived spillover FA across abdominal subcutaneous AT and found it to be considerably higher (*P* = 0.01) in women (28.5 ± 6.1%) than in men (9.9 ± 1.3%) [Fig. 3(b)].

### Associations between anthropometric, biochemical parameters, and chylomicron-derived spillover FA

**Figure 4.**
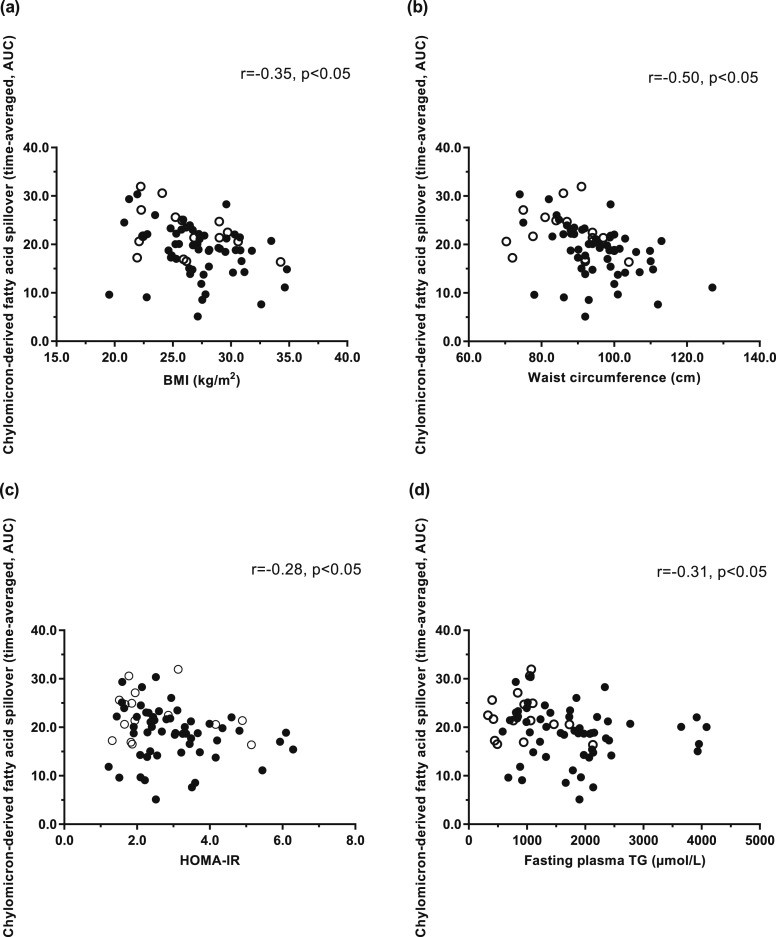
Correlations between the contribution of chylomicron-derived fatty acid spillover (time-averaged, AUC) to the plasma NEFA pool and (a) BMI (women: *r_s_* = −0.31; *P* = NS; men: *r_s_* = −0.33, *P* = 0.02); (b) waist circumference (women: *r_s_* = −0.36, *P* = NS; men: *r_s_* = −0.46, *P* = 0.0004); (c) HOMA-IR (women: *r_s_* = −0.23, *P* = NS; men: *r_s_* = −0.25, *P* = NS); and (d) fasting plasma TG concentrations (women: *r_s_* = −0.23, *P* = NS; men: *r_s_* = −0.22, *P* = NS) in women (n = 16) (white circle) and men (n = 55) (black circle).

We assessed the association between chylomicron-derived spillover FA anthropometric and biochemical variables for the whole cohort and found an inverse association among chylomicron-derived spillover FA (time-averaged, AUC), BMI (kg/m^2^) (*r* = −0.35, *P* < 0.05) [Fig. 4(a)], and waist circumference (*r* = −0.50, *P* < 0.05) [Fig. 4(b)]. We found no association between proportion of chylomicron-derived spillover FA (time-averaged, AUC) and fat mass (kg) (*r* = −0.20, *P* = 0.09) (data not shown). The proportion of chylomicron-derived spillover FA (time-averaged, AUC) was inversely associated with HOMA-IR (*r* = −0.28, *P* < 0.05) [Fig. 4(c)] and fasting plasma TG concentrations (*r* = −0.31, *P* < 0.05) [Fig. 4(d)]. Using a multiple stepwise linear regression model, we found that only sex and BMI were independently associated with chylomicron-derived spillover FA (sex, *r^2^* = 0.14, *P* = 0.05; and BMI, *r^2^* = 0.09, *P* = 0.01, respectively).

## Discussion

The spillover of chylomicron-derived FAs into the systemic NEFA pool has been suggested to occur because of the inability of AT to accommodate sufficient fat uptake; this may contribute to obesity-mediated insulin resistance as FAs are channeled toward ectopic fat depots (1, 3, 21). Using specific labeling techniques, we compared the proportion of chylomicron-derived spillover FAs appearing in the systemic plasma NEFA pool in men and women across a range of adiposity. We also had the opportunity, in a subgroup that had undergone arteriovenous difference measurements, with selective venous catheterization of subcutaneous abdominal AT, to explore the contribution of AT-specific chylomicron-derived spillover FAs in the plasma NEFA pool. We found chylomicron-derived FA spillover is influenced by degree of adiposity and sex; spillover was significantly higher in participants classified as nonobese compared with obese and in women compared with men.

Spillover of FA does not happen unless LPL is acting on TG-rich lipoproteins. The regulation of AT LPL is complex, with activity being low in the postabsorptive state and upregulated as part of an insulin-mediated response upon feeding (22); elevated plasma insulin concentrations and increased insulin secretion in the postprandial state have been associated with increased AT LPL activity (23). In the current study, individuals with a high BMI had significantly higher fasting, but not postprandial, plasma insulin concentrations than individuals with a low BMI. We found no association between the appearance of chylomicron-derived spillover FA in the systemic NEFA pool and postprandial plasma insulin concentrations. The appearance of chylomicron-derived spillover FAs in the plasma NEFA pool was significantly higher in participants with lower compared with higher BMIs, which may, in part, be explained by differences in the clearance of chylomicrons. It is plausible that the clearance of TG-rich particles is slower in individuals with a higher BMI because of a lower AT blood flow; there is a strong inverse association between AT blood flow and obesity (10, 24). A delay in the clearance of chylomicrons, resulting from a lower blood flow, would enable greater entrapment of LPL-generated FA in the AT. Our observations of a lower spillover in individuals with a higher compared with a lower BMI, along with the inverse association between spillover and BMI, align with reports that in obese AT there is a downregulation in the expression of genes in involved in lipid and FA metabolism pathways (10, 25). We did not observe an association between spillover and body fat, which may be due to differences in regional adiposity (*i.e.* the different functions in upper and lower body AT depots) and blood flow (11). Moreover, we cannot exclude that the higher chylomicron-derived FA spillover observed in the lower compared with higher BMI group was not influenced by the higher proportion of women in the lower BMI group. Although LPL activity is also mediated by a number of regulatory proteins, including apolipoproteins (26) and the angiopoietin-like proteins (27), it remains unclear how increased body fatness may influence these. AT LPL activity has been reported to increase when obese individuals decrease body weight and improve insulin sensitivity (28). However, Almandoz *et al.* (29) found no change in chylomicron-derived FA spillover after individuals with type 2 diabetes decreased their body weight by ∼14% (29). Taken together, and contrary to common suggestion (29, 30), the inverse association we observed here among spillover, BMI, and HOMA-IR would be indicative that high chylomicron FA spillover is a marker of good metabolic health.

Women have higher AT LPL activity compared with men (31, 32), with postprandial LPL activity in abdominal, gluteal, and thigh AT depots being greater in women (31). Sex hormones may influence AT LPL activity; plasma testosterone levels have been inversely correlated with abdominal and femoral AT LPL activity in sedentary obese men (33), whereas treatment with both estrogen and progesterone increased AT LPL activity, preferentially in the femoral depot, in postmenopausal women (34). We found the proportion of chylomicron-derived spillover FAs in the systemic NEFA pool was significantly greater in women compared with men and remained this way even after matching for plasma TG concentrations and HOMA-IR. In support of this finding are our observations from the substudy in which chylomicron FA spillover in subcutaneous abdominal AT was significantly higher in women compared with men. It has been suggested that women clear chylomicron-TG more rapidly than men (35); in this process, there would be a higher proportion of FAs entering the spillover pathway. Conversely, slower clearance of chylomicron-TG would provide AT with ample opportunities to store FA, thus resulting in less spillover.

It has been suggested that increased chylomicron FA spillover may lead to a greater FA flux and thus fat deposition in non-AT organs, such as the liver (3). However, tissue removal of chylomicron remnants via receptor-mediated uptake may also account for a considerable amount of TG delivery to the liver (6); this pathway is often overlooked as being a potentially important contributor to ectopic fat deposition. Studies tracing dietary fat over a 24-hour period have found that the contribution of FAs from chylomicron remnants to VLDL-TG, a surrogate for liver TG composition (36), are higher than the contribution of chylomicron-derived spillover FAs (36, 37). Thus it is plausible that chylomicron remnant TG plays a greater role in hepatic ectopic fat deposition than spillover FAs. A plausible reason for the lower contribution of spillover FAs to VLDL-TG is that a portion of the spillover FA are taken up into AT; we have previously reported that there is uptake of circulating NEFA into AT in the postprandial period (2).

Previous studies have used a dual tracer approach to determine the flux of chylomicron-derived spillover FAs (29, 30, 37–39). By using a continuous feeding regimen, a plateau in fat absorption rate is achieved along with steady-state chylomicronemia; in combination with a second systemic infusion (to quantify chylomicron-flux), fractional spillover resulting from LPL action could be determined (29, 30, 37–39). In the current work, we fed a single (labeled) mixed test meal and therefore cannot comment on the flux of chylomicron-derived spillover FAs resulting from LPL action. We found between 10% and 50% of the systemic plasma NEFA pool was derived from chylomicron-derived spillover FA, which is comparable to the values (20% to 50%) reported from dual tracer studies (29, 30, 37–39). The amount of fat ingested may influence the contribution of chylomicron-derived spillover FAs to the systemic plasma NEFA pool. Puga *et al.* (40) compared a low-fat (0.4 g/kg body weight) and moderate-fat (0.7 g/kg body weight) meal on postprandial spillover in males (63 to 71 years; BMI, 24 to 28 kg/m^2^) and found chylomicron-derived spillover was disproportionally increased at higher doses of dietary fat. This response was inversely related to adiposity (40), the latter being in line with the current study observation of decreased spillover with increased adiposity. Participants in the current study were fed a fixed amount of fat (40 g), independent of body mass, which equated to 0.55 g and 0.46 g fat per kilogram of body weight for women and men, and 0.58 and 0.45 g per kilogram of body for individuals with a low and high BMI, respectively. Despite women and low BMI individuals being fed more fat (per kilogram of body mass), they did not have a higher postprandial excursion in plasma TG; thus, it is plausible that chylomicrons were cleared more rapidly and in this process there is a higher proportion of FAs entering the spillover pathway.

There are some limitations to our study. We indirectly assessed the contribution of chylomicron-derived spillover FAs in the plasma NEFA pool and have assumed their appearance is derived from AT spillover. It is plausible that because of blood sampling timings and the postprandial period not being steady-state, we missed peak spillover, which has been reported to occur 4 to 6 hours after meal consumption (30). We cannot account for differences in chylomicron-flux and/or AT FA flux over the course of the study; therefore, we cannot exclude the possibility that spillover of FAs occurred at other nonadipose tissues such as skeletal muscle. We have previously suggested this contribution is most likely minimal (2). In the exploratory substudy, we directly assessed chylomicron FA spillover at the level of subcutaneous abdominal AT; however, because of the small number of participants, we had to make some assumptions and it is likely we did not have sufficient power to detect more robust changes. Moreover, we were unable to assess flux because we did not have sufficient data on AT blood flow. It would be of interest to assess flux and spillover in gluteal and thigh fat depots in a larger number of men and women across a range of adiposity. This would also provide the opportunity to compare tissue-specific and systemic calculations for chylomicron-derived spillover FAs.

Our data show that there is a considerable contribution of chylomicron FA that spillover into the systemic NEFA pool and that this process is considerably greater and more dynamic in lean individuals and in women. Contrary to general opinion, our findings suggest that spillover of chylomicron FA into the systemic circulation might not be a feature of the obesity-related metabolic complications, but rather the demonstration of good metabolic health.
